# Preventing Bone Loss in Breast Cancer Patients: Designing a Personalized Clinical Pathway in a Large-Volume Research Hospital

**DOI:** 10.3390/jpm14040371

**Published:** 2024-03-30

**Authors:** Inbal Dona Amar, Gianluca Franceschini, Camilla Nero, Ilaria Pasqua, Ida Paris, Armando Orlandi, Stefania Gori, Alessandra Fabi, Giorgia Garganese, Giovanni Scambia, Paola Villa

**Affiliations:** 1 Gynecologic Oncology Unit, Department of Woman, Child and Public Health, Fondazione Policlinico Universitario Agostino Gemelli IRCCS, 00168 Rome, Italy; camilla.nero@policlinicogemelli.it (C.N.); ilaria.pasqua@policlinicogemelli.it (I.P.); ida.paris@policlinicogemelli.it (I.P.); giorgia.garganese@policlinicogemelli.it (G.G.); giovanni.scambia@policlinicogemelli.it (G.S.); paola.villa@policlinicogemelli.it (P.V.); 2 Onco-Plastic Surgery Unit, Department of Woman, Child and Public Health, Fondazione Policlinico Universitario Agostino Gemelli IRCCS, 00168 Rome, Italy; 3 Scientific Directorate, Fondazione Policlinico Universitario Agostino Gemelli IRCCS, 00168 Rome, Italy; alessandra.fabi@policlinicogemelli.it; 4 Medical Oncology Unit, Department of Abdominal and Endocrine Metabolic Medical and Surgical Sciences, Fondazione Policlinico Universitario Agostino Gemelli IRCCS, 00168 Rome, Italy; armando.orlandi@policlinicogemelli.it; 5 Medical Oncology Unit, Department of Oncology, IRCCS Sacro Cuore “Don Calabria”, 37024 Negrar, Italy; stefania.gori@sacrocuore.it; 6 Rete Oncologica Pazienti Italia (ROPI), 20121 Milan, Italy; 7 Associazione Italiana di Oncologia Medica (AIOM), 20133 Milan, Italy

**Keywords:** breast cancer, clinical pathways, menopause, osteoporosis, patient care management

## Abstract

Background: We assess the impact of bone health clinical management in breast cancer (BC) patients receiving adjuvant endocrine therapy and design a personalized clinical pathway to reduce bone loss in an Italian research hospital. Methods: The primary endpoint was to assess (through the process improvement organizational method) the clinical pathway that post-surgical BC patients prescribed with endocrine therapy undergo to prevent bone loss. The secondary endpoint was to design a personalized clinical pathway for a prompt implementation of guidelines, to assess and possibly prescribe antiresorptive therapy. Results: During the first year of the execution of the new Diagnostic Therapeutic Assistance Pathway, a 60% increase in Dual-Energy X-ray Absorptiometry evaluations within 30 days and a 39.5% increase in antiresorptive therapy prescription within 90 days (since the prescription of endocrine therapy) were shown, thus increasing patients’ compliance. Conclusion: Case managers and bone health specialists in this context can improve patients’ adherence to therapies and bone health, helping physicians to expand their collaboration.

## 1. Introduction

Breast cancer (BC) is the most common diagnosed disease globally, with an estimated 2.26 million cases in 2020, and is the leading cause of cancer mortality among women [1]. In Italy, it represents 30% of all malignancies [2]. Mortality reached over 12,500 deaths in 2021, while survival 5 years after diagnosis was 88%. In 2022, over 55,700 new cases were diagnosed (+0.5% from 2020) [3]. Due to combined BC preventive and treatment strategies, patients’ overall survival is now standing at 80% in developed countries [4].

Since the most common subset of BC is hormone-receptor-positive [5], many patients receive adjuvant treatments with tamoxifen or aromatase inhibitors (AI) [6] with or without gonadotropin-releasing hormone (GnRH) agonists (depending on premenopausal or post-menopausal tumor onset) [7]. This adjuvant endocrine therapy results in a rapid deterioration of bone mineral density (BMD) and requires immediate prevention strategies [8].

Dual-Energy X-ray Absorptiometry (DXA) is currently a cornerstone radiological technique in osteoporosis diagnosis, playing a key role in stratifying the risk of osteoporotic fractures by assessing BMD [9]. The introduction of DXA scanners in 1987 to clinical practice marked a significant landmark that enabled precise osteoporosis diagnosis [10]. Radiation exposure doses of DXA examinations are significantly lower compared to conventional radiographic examinations and are comparable to background radiation levels [11]. The essential function of DXA remains the evaluation of BMD, necessary for identifying and monitoring the effects of osteoporosis treatment. DXA diagnosis of osteoporosis hinges on assessing the lumbar spine T-score of vertebrae L1-L4, as well as the T-score of the femoral neck and total proximal femur.

Poor bone health might result in fractures and other bone-related complications. To manage this issue, several international guidelines describe the proper implementation of treatments and prevention of bone disease in BC patients. An international joint position statement—published by several interdisciplinary cancer and bone societies—indicated specific T-score values, together with risk factors, to suggest bone-directed therapy for the duration of AI treatment to prevent fractures [8,12,13,14,15,16,17,18,19,20].

The anticancer benefits of adjuvant bisphosphonate treatment in postmenopausal women are represented by a 34% relative risk reduction in bone metastasis and a 17% relative risk decrease in BC mortality [8,21].

According to Hadji and colleagues, bone-addressed therapy should be administered to all patients with a T-score < −2.0, a T-score of <–1.5 SD with additional risk factors (age > 65; smoking; BMI < 20; family history of hip fracture; personal history of fragility fracture > 50 years; oral glucocorticoids use > 6 months), or ≥2 risk factors (without BMD) for the duration of AI treatment [8]. Patients with a T-score > −1.5 SD and no risk factors should be managed according to BMD loss during the first year and local guidelines for postmenopausal osteoporosis. Compliance should be assessed regularly, as should BMD treatment after 12–24 months. Furthermore, given the decreased incidence of bone recurrence and BC-specific mortality, adjuvant bisphosphonates are recommended for all postmenopausal women at significant risk of disease recurrence [8].

In 2020, guidelines from the Italian Association of Medical Oncology (AIOM) [22] suggested that bisphosphonates and denosumab were the drugs of choice in managing bone health in BC patients, as they have been shown to prevent BMD loss after adjuvant hormonal treatment. Direct efficacy against fractures has been demonstrated for denosumab 60 mg subcutaneous injections administered every 6 months in postmenopausal women undergoing AI therapy [23,24]. The AIOM stated that the optimal duration of treatment with bisphosphonates or denosumab in these women is not defined; however, it can be recommended to continue therapy at least for the period of treatment with GnRH and/or AI drugs. In a broader perspective, ensuring adequate levels of vitamin D and calcium through diet or supplementation is essential to support antiresorptive bone therapy; the daily calcium requirement for menopausal women is 1500 mg, while the average recommended intake of vitamin D is 400–800 IU/day.

Similarly, in 2017, the Italian Drug Agency (Agenzia Italiana del Farmaco, AIFA) published a document known as “Note 79” stating that primary prevention with bone-modifying agents, such as bisphosphonates or denosumab, is necessary in menopausal women at increased risk of fracture following adjuvant hormonal blockade treatment for BC. The intake or supplementation of vitamin D and calcium in the diet is also recommended [25].

Considering the abovementioned guidelines and clinical trials [24,26,27,28] supporting the use of bisphosphonates or denosumab in BC patients undergoing AI therapy to prevent bone loss, it is important to understand how such recommendations are followed within specific clinical scenarios. For example, a study by Recine et al. assessed the real-life clinical impact of bone health in patients with BC receiving adjuvant endocrine therapy in an Italian Osteoncology Center (research hospital), confirming that bone health management is an essential part of long-term cancer care [29].

In our retrospective study, we assessed the impact of bone health clinical management in post-surgical BC patients receiving adjuvant endocrine therapy in an Italian large-volume research hospital. The primary endpoint was to analyze the clinical pathway that post-surgical BC patients with a prescription of AI treatment undergo to prevent bone loss. The secondary endpoint was to design a personalized clinical pathway to ensure a prompt implementation of international and local guidelines, for the assessment and possible prescription of antiresorptive therapy (with bisphosphonates or denosumab) with the goal of primary or secondary prevention.

## 2. Methods

Research hospitals are essential institutions specialized in the diagnosis, treatment, and prevention of diseases by combining research and care. They serve as the hub of biomedical sciences, where clinicians and researchers work together to develop excellent treatments, devices, and therapies. In 2018, the Italian Ministry of Health recognized our polyclinic as a research hospital for the disciplines of “Personalized Medicine” and “Innovative Biotechnologies”, aimed at providing cutting-edge research and advanced treatment options to patients from around the globe. In 2021, our research hospital received the Joint Commission International (JCI) accreditation and, in 2022, we joined the Organization of European Cancer Institutes (OECI) network.

One of the unique features of our institution is a strong commitment to precision medicine research, which aims at finding “the right treatment, to the right patient, at the right time”, [30] allowing us to tailor and develop innovative treatments unique for the specific needs of our patients. To this end, our hospital has a state-of-the-art research infrastructure that houses over 20 laboratories (e.g., biobank, bioinformatics, organoids, 3D bioprinting, genomics, radiomics, liquid biopsy, big data analysis) to support our scientists in conducting their research according to the highest quality standards.

For example, BC biological tissues can be stored in our biobank after surgery and therefore studied for multi-omic profiling, patients’ stratification, and deep phenotyping [31]. This research infrastructure allowed for the implementation of a Comprehensive Cancer Genome Profiling Program for 10 oncological diseases (including BC); such a hybrid program combines clinical practices and translational research by using a 500-gene panel to acquire mutational information on tumor tissue, allowing for the identification of genome alterations that may respond to current target therapies while extending the possibility for patients to access novel treatments, thereby providing targeted therapies [32]. Specifically, the project for the genomic profiling of BC patients started in 2022, and to date, 11 patients have been profiled.

In this scenario, we conducted an organizational analysis to understand how specialists involved in the care of post-surgical BC patients (prescribed with AI treatment) manage their risk of bone loss, define the workflow status, and eventually, increase its quality by redesigning the model. The new clinical pathway is part of the study “HEQUOBIP—health and quality of life in Oncological patients: management of bone pathology in the Italian cancer treatment-induced bone loss (CTBL) population” (PROT 5147/18 ID 18—Clinicaltrial.gov: NCT04055805) and received the approval as a standard clinical pathway (PCA—Pathway of Clinical Assistance) for breast cancer on 16 June 2022.

Our institution is a research hospital, and this pathway (among others) adheres to national regulations, institutional policies, and the Helsinki Declaration. Approval was granted by the Ethics Committee of the hospital (Fondazione Policlinico Gemelli IRCSS in Rome (Italy) [Approval ID:1893]. Patients provide prior written consent before undergoing any clinical procedure and are treated according to international guidelines. To redesign the pathway, we referred to the process improvement organizational method [33,34], which consists of four phases, described as follows:

1. Analysis: process mapping (“as is”), goals assessment, identification of priorities and key processes, and creation of the team.
2. Planning: identification of gaps and critical points and definition of the most relevant changes.
3. Action: change implementation, definition of key performance indicators, and development of a data collection method.
4. Monitoring: control and plan of future improvements and integration with the management control process.

The Analysis phase explores currently conducted activities and the value of the current activities in the clinical pathway of BC patient candidates for antiresorptive treatments. Oncologists, gynecologists, breast specialists, and surgical radiotherapists at our research hospital created a team to assess the workflow by identifying its weaknesses and potential. Considering its limitations and strengths, we designed a personalized clinical pathway (Planning) to enable the prompt implementation of antiresorptive therapy (according to guidelines) to prevent bone loss and fragility fractures by considering unmet needs and introducing changes in the workflow (Action), with the ambition of reducing BMD assessment to within 30 days and therapy prescription to within 90 days (Monitoring).

## 3. Results

### 3.1. Analysis and Planning

**Figure 1:**
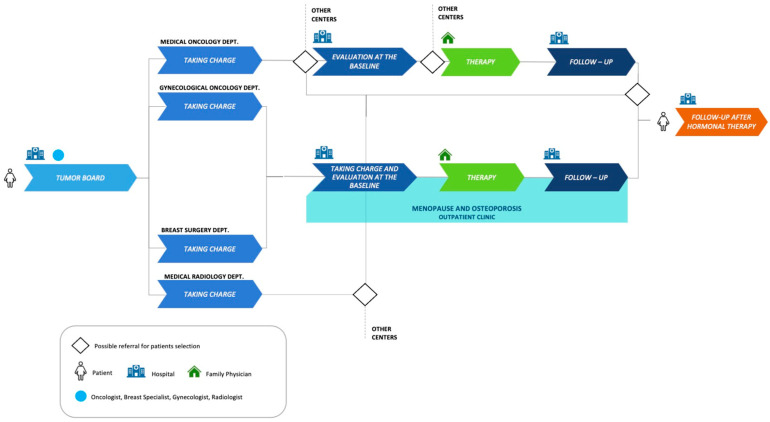
Previous Clinical Pathway. Flow diagram describing the clinical pathway for breast cancer patients at our research hospital until 2021.

In our previous model (Figure 1), post-surgery BC patients were discussed by a multidisciplinary tumor board, and those with hormone-receptor-positive tumors received indications to start adjuvant endocrine therapy. These patients continued their clinical course in one or more medical departments (Medical Oncology, Gynecological Oncology, Breast Pathology, and Medical Radiology). Some of them were referred to our outpatient clinic specialized in menopause and osteoporosis for a basal evaluation of bone status, including Dual-Energy X-ray Absorptiometry (DXA) of the lumbar spine and the hip and a panel of bone metabolism blood tests, followed by the prescription of antiresorptive treatment, if appropriate.

Patients underwent annual follow-ups with hip and spine DXA and a serum bone metabolism profile until endocrine therapy ended. A final follow-up was scheduled at the end of the AI treatment. The rest of the patients (not referred to our menopause and osteoporosis outpatient clinic) were referred to different bone health centers nearby or within our hospital.

In analyzing the previous pathway, several critical points and unmet needs were identified. Initially, there was a mismatch between different specialists due to different working hours and organization. The resulting delay in evaluation led to inconsistencies in the treatments prescribed (patients were disoriented by the different professionals approaching the problem).

Regarding the patient’s taking charge phase, the presence of a tumor board that defines treatment strategies for each patient represented a key asset. Limitations consist of the absence of defined criteria for the distribution of patients between professionals and departments. We, therefore, understood that criteria should be defined and a bone specialist (e.g., gynecologist, rheumatologist, orthopedic, or endocrinologist) should be identified and involved as early as the tumor board stage so that the bone health pathway could be defined at the same time as the therapeutic strategy.

Another limitation of this phase consisted of the lack of implementation of a structured bone pathway, which assesses bone status and treats it according to current shared guidelines, at the time of the prescription of endocrine treatment during the tumor board review.

During the basal assessment phase, the presence of an outpatient clinic for menopause and osteoporosis within the hospital represented a strength. On the other hand, limitations consisted of the lack of dedicated slots for the required diagnostic examinations and the absence of shared assessment criteria for patients’ bone status at the baseline. We also found discrepancies in the timing of patient assessment between different professionals, while the absence of a key case manager in such a pathway represented a major weakness.

In the treatment phase, a strength was noted regarding the involvement of the family physician in prescribing denosumab after receiving the treatment plan from the specialist. It was also noted that patients were educated to adopt a correct lifestyle, eat healthily, and perform physical activity. Limitations included the lack of a multidisciplinary team in prescribing treatment and the absence of the implementation of Note 79.

**Figure 2:**
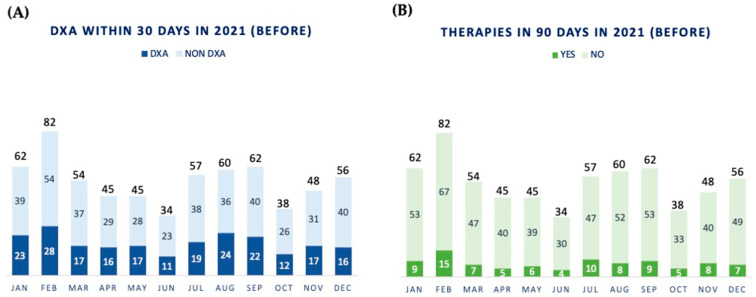
Preliminary data regarding the old clinical pathway’s performance. Number of DXA sessions assessed in 30 days (**A**) and number of antiresorptive therapies prescribed in 90 days (**B**) to breast cancer patients. Workflow of the former clinical pathway in 2021.

In 2021, n. 643 patients with a new diagnosis of BC were discussed in our multidisciplinary tumor board reviews, 34% (n. 222/643 patients) of whom underwent DXA evaluation within 30 days (Figure 2A). Only 14% (n. 93/643 patients) were prescribed antiresorptive therapies within 90 days (Figure 2B).

Regarding the follow-up phase, DXA and serum profiles of bone metabolism markers were performed annually, and if there was any change in bone status, the patient was referred to our menopause and osteoporosis outpatient clinic or to the oncologist; even in this case, we pointed out the lack of a case manager to coordinate the bone health pathway. Another limitation was the discrepancy among oncologists regarding the application of eligibility criteria for Note 79 and the timing of bone health assessment.

Additionally, some unmet needs were identified, including the lack of shared criteria between departments for evaluating whether to continue or not treatment after the end of adjuvant endocrine therapy. The lack of referring patients to dedicated bone specialists and the absence of structured protocols for other hospital specialists were noted. The need for a case manager to support the bone health pathway (e.g., filling out the treatment plan, helping manage the follow-up phase, and contributing to patient education) remained a primary issue.

Considering all these challenges, the design of a new personalized clinical pathway seemed the most appropriate organizational choice. This analysis created an opportunity to generate a defined workflow that promotes specialists’ training in and awareness of defining activities, responsibilities, and timelines focused on the proper implementation of therapeutic choices.

### 3.2. Action and Monitoring

As we understood there was a need for implementing a novel pathway to systematically treat patients in a more personalized manner, we also set the goal of substantially reducing BMD assessment to within 30 days and therapy prescription to within 90 days. Such a project required the introduction of the following organizational changes:

1. Alignment between the professionals and the design of a dedicated, transversal bone health pathway to be applied for all BC patients undergoing hormonal adjuvant therapies, in line with Note 79 (regarding the correct therapy definition) for personalized patient management and care.
2. Identification and integration of a bone specialist from the tumor board phase, to define every therapeutic strategy for each patient and evaluate the treatment of bone loss.
3. Introduction of a case manager (nurse) as a pivotal figure for interprofessional coordination, supporting therapy prescription and follow-up, as well as patient education.
4. Training the health professionals and administrative staff involved in activity ownership and timing, to select the best care treatment and facilitate the organizational workflow.

**Figure 3:**
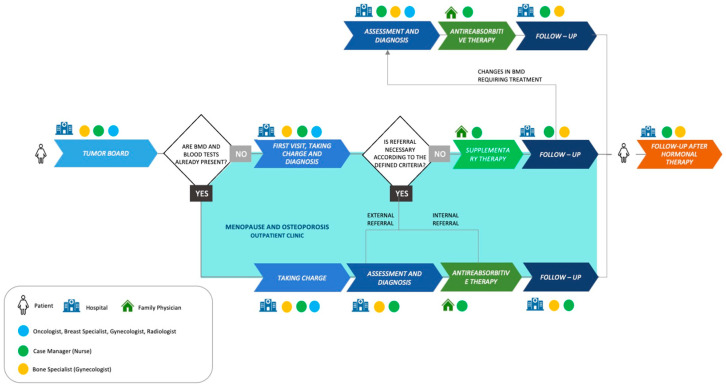
New Clinical Pathway. New Diagnostic Therapeutic Assistance Pathway for breast cancer patients at our research hospital.

We designed a Diagnostic Therapeutic Assistance Pathway (DTAP) that fully implements international and local guidelines after the verification of correct therapies, along with a structured bone health treatment pathway for patients (Figure 3). The pathway includes a worktable with defined criteria presenting the treatment guidelines, to update the participating specialists. It was designed according to the value-based healthcare model, a patient-centered transversal approach to organizational processes [34].

During the tumor board review, a multidisciplinary team consisting of gynecologists, breast specialists, radiologists, and oncologists follows a shared protocol that defines the diagnostic procedures required for the basal assessment of bone health, the timing of these assessments, and the criteria for interpreting the diagnostic results. For each case discussion, if a decision is made to prescribe adjuvant endocrine treatment, the bone health pathway is activated by the case manager (in our organization, this is a nurse). Patients’ information is inserted into a computerized database for registration. The case manager then creates an Excel file containing the patient’s details and email addresses. Prior to each stage of the pathway, patients are contacted via phone and receive a comprehensive email including communications that outline the upcoming steps and provide clarity on their role in the procedure. The case manager verifies, for every BC patient who is a candidate for adjuvant endocrine treatment, whether she has been evaluated by a bone specialist (in our organization, this is a gynecologist):

(a) If the patient has not been evaluated, the case manager schedules an appointment within 30 days of the prescription of adjuvant endocrine treatment.
(b) If the evaluation is already present, the case manager assesses whether the examinations are recent and have them repeated if necessary.
(c) If no such evaluation is present, the case manager prescribes DXA of the spine and femur and a blood panel for bone metabolism assessment.

Once the patient has completed the tests, the case manager schedules a meeting at our menopause and osteoporosis outpatient clinic, where the patient is evaluated by the bone specialist. 

This evaluation and eventual treatment take place within 90 days of the start of adjuvant endocrine treatment. The bone specialist evaluates diagnostic tests and, taking into account the patient’s personal history, risk factors, Note 79, and other medical guidelines, prescribes antiresorptive treatment, and, if appropriate, vitamin D and calcium supplementation. Bone health status is monitored by DXA and blood tests throughout the duration of adjuvant endocrine treatment.

**Figure 4:**
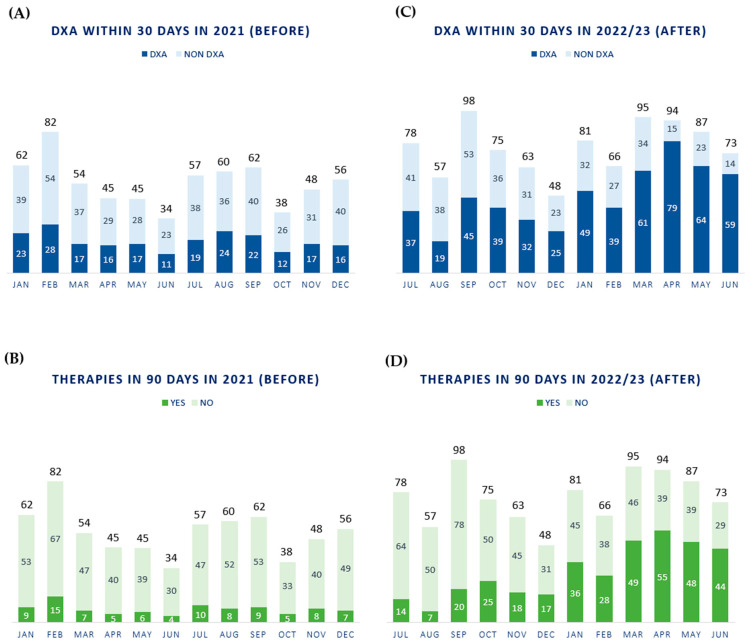
Comparison between the performances of the old and new clinical pathways. Comparison in the performances achieved with the old clinical pathway (**A**,**B**), and the new Diagnostic Therapeutic Assistance Pathway (**C**,**D**).

At the end of the BC therapy, further assessment of bone health is conducted to decide whether antiresorptive therapy should be continued, modified, or suspended. In the case of vitamin D supplementation alone following normal BMD, the patient will continue annual follow-ups to monitor any decline in BMD status. After this evaluation, patients continue to follow-up if needed. In this scenario, we monitored the first year of the new DTAP (Figure 4A–D).

From July 2022 to June 2023, n. 915 patients were discussed in our multidisciplinary tumor board for breast cancer, 60% (n. 548/915 patients) of which underwent DXA evaluation within 30 days, and 39.5% (n. 361/915 patients) were prescribed antiresorptive therapies within 90 days, thus increasing patients’ compliance. The mean age of the patients was 53.2 years old, and the mean BMI was 23.7. Forty-eight percent of the patients were treated by chemotherapy, while 71.5% underwent radiotherapy. All patients received the standard therapy for the stage of disease with AI with or without ovarian suppression, depending on menopausal status.

## 4. Discussion

In complex healthcare scenarios, the standardization of procedures and practices is not always feasible for several reasons, including patient flows (in terms of volumes of activities), human resources, organizational mission and vision, clinical assets, and expertise [34]. However, our experience could be useful, allowing us to acknowledge some aspects that can facilitate patient management in large-volume hospitals, with the specific goal of preventing bone loss in BC patients. In fact, the preliminary results shown in Figure 4C,D seem to be promising for our goal of substantially increasing the frequency of DXA assessments within 30 days and ensuring therapy is prescribed within 90 days after AI treatment prescription.

In our research hospital, the case manager contributed significantly to coordinating numerous activities in the new personalized clinical pathway. Case managers possess skills in assessment, monitoring, cultural competence, interpersonal collaboration, and coordination, and they advocate for resources and services to achieve patient goals in the health industry. Such professionals also address other social determinants of health, such as the patient’s socioeconomic status, literacy, income, employment, and working conditions that affect patients’ health [35]. As a nurse (a professional with several skills related to interprofessional education [36]), our case manager facilitated communication between different specialists and departments, promoting the exchange of clinical information among them efficiently. The case manager also helped in reducing patient disorientation by coordinating activities, workflows, and time schedules.

From a clinical perspective, the integration of the case manager into the new pathway (Figure 4C,D) helped in reducing patient dropout due to the dispersion of the previous workflow (Figure 4A,B). It also improved patients’ compliance with treatments by promoting their education while providing information and counseling. Therefore, case managers in bone healthcare can be of paramount importance in harmonizing the organizational workflow and conducting activities (e.g., monitoring the patient’s DXA, and scheduling future visits and diagnostic tests).

Another crucial role is played by the bone specialist who followed all cases and joined the patient’s case from the tumor board stage. Bone specialists specialize in the diagnosis, treatment, and management of osteoporosis and other metabolic bone diseases, providing rehabilitation and prevention measures to improve patients’ bone health [37]. Thus, collaboration among specialists from different departments enriches knowledge and keeps specialists up to date with current guidelines regarding bone pathology. Communication among them was facilitated by the presence of the case manager on the tumor boards. Their cooperation was significant for developing a shared protocol and defining common interpretative criteria for diagnostic results.

In 2022, Italy was recognized as the country with the second-greatest percentage of aging adults (23%) after Japan (28%) [38]. As the population gets older and treatments for breast cancer increase survival, we will treat more patients at risk of bone loss and fractures in the future. Untreated bone loss often worsens, generating fractures and disabilities requiring surgery and, at times, the insertion of prostheses. Our results show that the implementation of a new DTAP increased the number of patients undergoing specific bone quality analysis within 30 days after the tumor board review (60% compared to the former 34%) (Figure 4A,C, respectively). It also increased the number of patients that were prescribed antiresorptive therapies within 90 days after the tumor board review (37.5% compared to the former 14%), thus improving the promptness of treating bone loss (Figure 4B,D, respectively). Such findings are important for the clinical prevention of fragility fractures and the treatment of bone pathology in these patients, personalizing “the right treatment for the right patient, at the right time”.

According to Cauley [39], the major consequences of bone fractures consist of increased mortality for hip fractures, functional consequences (such as reductions in quality of life), and economic burden. Given the high impact of this social phenomenon on health systems worldwide, we outline the importance of fully integrating bone specialists and case managers in clinical pathways from the tumor board stage, to diagnose and treat bone loss early, according to local and international guidelines.

Since the project was developed in a research hospital, we must recall that the clinical pathway is strictly intertwined with research laboratories and organizational strategies. The development of an appropriate pathway of care could lead researchers to enroll patients in clinical trials and perform translational medicine studies. Artificial intelligence algorithms can help identify patients eligible for clinical trials by matching medical records with inclusion criteria to verify eligibility for enrollment [40]. The next steps will include collecting data on patients discussed at tumor board reviews to longitudinally assess how the new clinical pathway improves multidisciplinary clinical conduct. Future research in this clinical pathway will assess the molecular and genetic characteristics of patients with the aim of finding precise biomarkers for preventing bone loss.

## Data Availability

Data are contained within the article.
